# In this month’s Bulletin

**DOI:** 10.2471/BLT.26.000126

**Published:** 2026-01-01

In the editorial section, Arham Kamil and Abdur Rafay Farooq (2–3) advocate for better care for survivors of emergencies. Olive Cocoman et al. (4–5) call attention to the specific needs of mothers and their babies during humanitarian crises.

## China 

### Reduction of non-prescription antibiotic use 

Minzhi Xu et al. (28–38) do a randomized trial of a family doctor-led intervention. 

## Democratic Republic of the Congo, United Republic of Tanzania

### Treatment of malaria in children

Samwel Gesase et al. (17–27) compare artesunate regimens.

## Ethiopia

### Universal health coverage targets

Yibeltal Assefa et al. (8–16) analyse progress towards 80% coverage. 

## Indonesia

### Protocol deviations and medicine tests

Esti Mulatsari et al. (39–46) outline difficulties with the use of standard testing methods. 

## Global

### Antibiotic classifications

Veronica Zanichelli et al. (47–49) examine limitations of reserve listings. 

### Diagnosis of diabetes 

Bianca Hemmingsen et al. (50–52) quantify gaps in testing and treatment.

### Global action plan on antimicrobial resistance

Mathieu JP Poirier et al. (53–55) propose the use of social science to improve problems definitions.

